# PulseRider Aneurysm Neck Reconstructive Device Versus Other Treatments in Patients With Wide-Neck Bifurcation Aneurysms: A Systematic Review

**DOI:** 10.7759/cureus.87479

**Published:** 2025-07-07

**Authors:** Mariana Olvera Morales, Diego Santillán Alcántar, Jose R Flores Valdés, Mariana Del Rio Rocha, Mark Reyes Anaya, Mauricio Montelongo Quevedo, Karla Y Bujdud Rodríguez, Gilberto Granados García, Luis C Lora Ponce, Sergio A Villar López, Juan P Navarro Garcia de Llano

**Affiliations:** 1 General Medicine, Universidad Autónoma de Guadalajara, Guadalajara, MEX; 2 General Surgery, Instituto Mexicano del Seguro Social Regional General Hospital No. 46, Guadalajara, MEX; 3 General Physician, Oncology Consultants, P. A., Houston, USA; 4 General Medicine, Universidad Autónoma de Yucatán, Mérida, MEX; 5 General Medicine, Instituto de Estudios Superiores de Tamaulipas, Red de Universidades Anáhuac, Miramar, MEX; 6 General Medicine, Universidad de Guadalajara, Guadalajara, MEX; 7 Neurological Surgery, Mayo Clinic Jacksonville, Jacksonville, USA

**Keywords:** aneurysm, bifurcated, equipment, neck, supplies, wide neck

## Abstract

Wide-neck bifurcation aneurysms (WNBA) are challenging to treat both surgically and endovascularly due to their complex anatomy. The FDA-approved PulseRider Aneurysm Neck Reconstructive Device (PRANRD; Cerenovus, Johnson & Johnson, Irvine, CA, USA) offers a novel endovascular treatment option; however, comparative data on its safety and efficacy remain limited. This review compares PRANRD with other treatment modalities for unruptured WNBA. A search of multiple databases identified 51 articles, of which three met the inclusion criteria, comprising two cohort studies and one case series, for a total of 134 patients treated with PRANRD. The device achieved complete angiographic occlusion in most cases with low complication rates. Compared to Y-stenting, PRANRD-assisted coiling demonstrated similar immediate occlusion rates but lower complete occlusion rates at six months. No procedure-related mortality was reported. However, long-term follow-up and randomized controlled trials are still required. PRANRD appears to be a promising alternative for treating WNBA, offering high occlusion rates and a favorable safety profile. Further research is necessary to confirm its long-term efficacy and safety.

## Introduction and background

An aneurysm is an abnormal focal dilatation or bulge in an artery due to the intrinsic weakness of the vessel wall [1,2]. They are often asymptomatic and typically undetected. The development of an aneurysm occurs during adulthood, and growth is associated with risk factors such as age, hypertension, pre-existing familial conditions, and smoking [3]. These dilations are classified according to their shapes. Most aneurysms are saccular and are anatomically classified into two groups: sidewall and bifurcation aneurysms. Saccular unruptured intracranial aneurysms affect 3-5% of the adult population, irrespective of geographical location or ethnicity. They develop after the second decade of life, most often between the fourth and sixth decades, and are more prevalent in women than men. The overall prevalence is estimated to be 3.2% in a population without comorbidities [1,4,5]. Wide-neck bifurcation aneurysm (WNBA) is defined as an aneurysm that has a neck of >4 mm or a dome-to-neck ratio of <2, and they are among the most difficult saccular aneurysms to treat [6,7]. The particular anatomy of a wide-neck aneurysm makes its treatment through surgical or endovascular modalities challenging [8]. Treatment methods include two major intervention options: clipping of the aneurysm and endovascular methods such as coiling, stent-assisted coiling, flow diversion stents, or balloon remodeling. Although intracranial aneurysms have multiple management options, recent trends favor endovascular treatments, as they are considered safer and a more effective alternative [3,9,10].

PulseRider Aneurysm Neck Reconstructive Device (PRANRD) (Cerenovus, Johnson & Johnson, Irvine, CA, USA) is a newly FDA-approved bifurcation intracranial aneurysm device. The device consists of a permanent nitinol (nickel-titanium) self-expanding stent implant for treating wide-necked aneurysms located at or near branching areas of arteries in the brain [11]. The stent configuration comes in a Y- or T-shape and is delivered via a standard microcatheter with an inner diameter of 0.021 inches. The device is retrievable and may be repositioned by retracting it into the microcatheter at any time during or after deployment. It is deployed at the parent vessel bifurcation and across the aneurysm neck to provide a supporting framework, bridging the aneurysm neck while retaining coils within the aneurysm [12]. It is specifically designed to preserve luminal patency and maintain hemodynamic flow through the parent vessel bifurcation while minimizing exposed metal, thereby encouraging early endothelialization and securely retaining coils within the aneurysm sac [13].

Few studies in the literature have compared multiple treatments, mainly endovascular and microsurgical. Advances in endovascular techniques have been designed to accommodate variations in anatomy. However, there is a lack of studies examining the efficacy and safety of PRANRD, comparing its outcomes to those of numerous other endovascular treatments. This systematic review aims to compare the effectiveness and safety of PRANRD with different available surgical and endovascular modalities used in unruptured (WNBA).

## Review

Methods

Search Strategy

This systematic review adheres to the guidelines established by the Preferred Reporting Items for Systematic Reviews and Meta-Analyses (PRISMA) 2020. It follows the methodological recommendations outlined in "10 Steps to Conduct a Systematic Review" by Calderón-Martínez et al., both of which are available as open-access resources under the Creative Commons Attribution license [14,15]. With registration officially obtained and validated through PROSPERO with the ID CRD42024571372.

We conducted a systematic review of the literature to evaluate the PRANRD in comparison to other treatments for patients with WNBA. This device is relatively new, and our research spans the last three years. We accessed various databases, including PubMed and ScienceDirect. We applied a combination of keywords, including "wide neck", "bifurcated", "aneurysm", "neck", and "equipment and supplies", to gather the necessary information for this study. Accessible text terms and Medical Subject Headings (MeSH) were applied on August 29, 2024.

*Types of Study*

To investigate PRANRD, we conducted a systematic review of the applicable studies. They were published from 2017 to 2020 and are available in English. We thoroughly screened and analyzed cohorts and case series, as well as studies that met our inclusion criteria, as disclosed on PRANRD. Our study focused on patients with unruptured wide-neck aneurysms who were treated with the device. This included cases where other endovascular techniques were deemed inadequate, and PRANRD was considered a viable alternative treatment.

Case reports, cross-sectional studies, book chapters, protocol articles, reviews, conference abstracts, letters to the editor, comment publications, and dissertations were excluded from this study. In addition, cases with ruptured WNBA were excluded from this systematic review. Moreover, studies that did not detail their methodology, duplicates, and those from which we could not obtain the required data or receive a response from the original author via email were also excluded.

Types of Participants

This study established specific criteria for participant selection, including individuals of all genders and those aged 18 and over, to ensure a diverse sample for a comprehensive understanding of the intervention.

Patients who underwent anticoagulant or antiplatelet therapy during the perioperative period for unruptured WNBA were also included in the study. The cerebral angiography findings encompassed anatomical sites of intra- and extracerebral arterial bifurcations, including the terminal carotid artery, basilar apex, anterior cerebral artery, middle cerebral artery, and posterior cerebral artery. Studies involving pediatric patients, pregnant patients, and patients with a history of ruptured WNBA were omitted.

*Types of Intervention*

To qualify for inclusion in this study, the selected research needed to assess the safety and efficacy of PRANRD in adult patients with WNBA. The control group could consist of individuals who received endovascular surgical treatment. Studies that addressed a non-endovascular approach were excluded.

Outcomes

The primary outcomes analyzed in this study included treatment-related factors, such as stability and efficacy, as well as significant complications, including distal vessel perforation and aneurysm perforations. These complications were assessed explicitly through measures of neurological impairment, using the modified Rankin scale (mRS), as well as the risk of ischemic or hemorrhagic events.

Secondary outcomes focused on morbidity and mortality, particularly the failure of treatment, which was evaluated based on suboptimal device positioning. This was measured using a 3-point scale that includes total arterial occlusion, neck remnant, and aneurysm remnant.

Selection of Studies

Following an initial screening based on the title and abstract, two reviewers (KYBR, MRR) independently selected trials for inclusion in this review using predetermined inclusion and exclusion criteria. Disagreements regarding study inclusion were resolved through consensus and consultation with a third review author (MMQ). For full-text screening, two reviewers (KYBR, MRA) independently selected trials for inclusion in this review using predetermined inclusion and exclusion criteria.

Assessment of Risk of Bias in Included Studies

For the evaluation of the two cohort studies, we used the Newcastle-Ottawa Quality Assessment Scale (NOS) [16]. Two independent reviewers (DSA, GGG) assessed the included articles based on the specified criteria and evaluated each component, and then the evaluation was carried out. The results were interpreted in light of the NOS discrepancies between the initial reviewers, and these discrepancies were resolved by incorporating a third reviewer (LCLP), who also followed the guidelines and classified them as good quality, fair quality, or poor quality.

For the evaluation of the case series study, we utilized the Joanna Briggs (JBI) Checklist for Case Series [17]. This assessment was also carried out by two independent reviewers (DSA, GGG), with any discrepancies resolved by a third reviewer (LCLP).

Results

**Figure 1:**
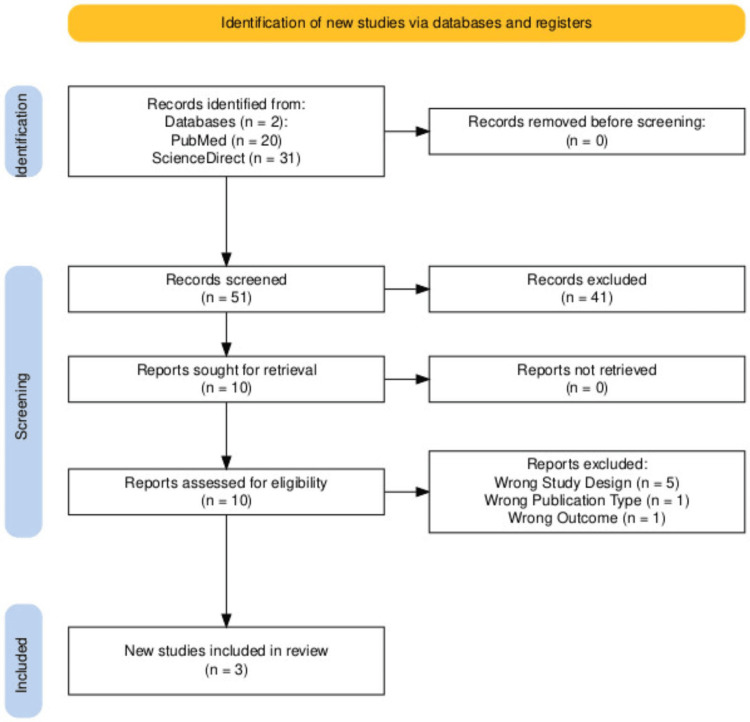
PRISMA flowchart Fifty-one records were screened. Records were identified, screened, excluded, sought for retrieval, assessed for eligibility, and excluded for the following reasons: five articles had an incorrect study design, one article had a wrong publication type, and one article had a wrong outcome [14]. PRISMA: Preferred Reporting Items for Systematic Reviews and Meta-Analyses

Our study selection, as shown in the PRISMA flow diagram, initially yielded 51 records. After removing duplicates, screening titles, abstracts, and other types of reviews that were not cohort studies and case series, 10 full-text articles were eligible. Ultimately, three studies met our inclusion and exclusion criteria and were selected for this review (Figure 1).

The PRISMA flow diagram summarizes the characteristics of the three studies included in our analysis. Initially, seven articles were identified; however, after applying various filters to refine the data and relevance, we narrowed the selection to three articles that aligned with the objectives and requirements of our study. The majority of the studies included were cohort studies (n = 2), accompanied by one case series (n = 1).

For the evaluation of the risk of bias, the studies were categorized into two groups: case series and cohort studies. Case series were evaluated using the JBI Checklist for Case Series, which is freely available as an open-access tool from the JBI website. Cohort studies were assessed using NOS, which is publicly available for research use but not formally published under an open-access license. Proper citation and acknowledgment have been provided for both tools [16,17]. Our selection comprised three articles, all of which were of good quality (100%), with none rated as fair quality (0%) or poor quality (0%). The detailed results outline the criteria used to determine the classification of each study, which are summarized in Table 1.

**Table 1:** NOS for the risk of bias appraisal of cohort studies Three studies were evaluated using NOS. The results were categorized as good quality, fair quality, and poor quality according to the following criteria: Good quality studies were defined as those with 3 or 4 stars in the selection domain, 1 or 2 stars in the comparability domain, and 2 or 3 stars in the outcome/exposure domain. Fair quality studies: 2 stars in the selection domain, 1 or 2 stars in the comparability domain, and 2 or 3 stars in the outcome/exposure domain. Poor quality studies: 0 or 1 star in the selection domain, 0 stars in the comparability domain, and 0 or 1 star in the outcome/exposure domain [16]. NOS: Newcastle-Ottawa Quality Assessment Scale

Author, year	Study design	Selection	Comparability	Outcome/exposure	Total	Subjective evaluation
Limbucci et al., 2020 [18]	Cohort	3	1	3	7	Good quality
Gory et al., 2017 [19]	Cohort	3	2	2	7	Good quality

One case series was included [20]. The methodological quality of the study was assessed using the JBI tool for case series [17]. The key findings are as follows: the inclusion criteria for the studied population were clearly defined as unruptured WNBA located at the basilar tip, terminal carotid artery, anterior cerebral artery, or middle cerebral artery, as determined by cerebral angiography. Cerebral angiography was appropriately utilized as a valid method for identifying the pathological condition of the patients included in the case series. Although it was not specified whether the patients were consecutive cases, it is noted that all patients included in the study were followed for six months. The study provided incomplete demographic data. While information such as age and gender were included, details like ethnicity, education, and the geographic location of each participant were not provided. The clinical condition of the patients was described in detail, including disease presentation, family history, comorbidities, and previous interventions. The intervention performed was described with precision, including details of any complementary procedures. The clinical condition of the patients and follow-up after the intervention were thoroughly documented through immediate post-intervention angiography and a six-month follow-up, as well as the use of the mRS, which was recorded prospectively.

Overall, the methodological quality of the case series study included in this systematic review is deemed satisfactory. Most of the critical elements required for robust methodological quality are present, although a key limitation is the incomplete demographic data. This limitation should be taken into account when interpreting the findings and their potential applications.

The studies included in this review encompass both cohort studies and case series, providing a comprehensive perspective on the clinical outcomes associated with this innovative device, which operates across multiple countries [14-16].

Results of Individual Studies

Limbucci et al. conducted a retrospective cohort study in Italy. Participants had a mean age of 56.47 ± 11.54 years, with hypertension being a common comorbidity. The study enrolled 105 patients; 73 patients of the control group were treated with Y-stenting, and 32 patients were treated with PRANRD. In the angiographic results, immediate occlusion, as graded by the Raymond-Roy scale, was successful in 22 cases (RR1), in nine cases (RR2), and in one case (RR3) of the intervention group. Moreover, in the control group, immediate occlusion was classified as RR1 in 61 instances, RR2 in eight cases, and RR3 in four cases. Overall, adequate occlusion was achieved in 31 patients with PRANRD and 69 patients in the control group. At the six-month follow-up, overall occlusion was achieved in 27 patients with PRANRD and 67 patients in the control group.

Complications were reported in two cases within the intervention group, including one ischemic complication due to intraprocedural branch thrombosis, which was managed with tirofiban without clinical sequelae, and a case of dysphasia and hemiparesis that occurred one day after the procedure. Furthermore, the control group reported six complications, including one distal vessel perforation, three aneurysm perforations determining subarachnoid hemorrhage, one patient with perforation had a minimal residual disability, and one patient with aneurysm perforation died after subarachnoid hemorrhage and ventricular drain infection (mRS6). Mortality was 0 cases in the intervention group and one case in the control group [18].

Mukherjee et al. published a case series based in the UK. Patients with a mean age of 55 years were considered, and PRANRD was used. Results showed 10 successful cases, which remained successful at the six-month follow-up. There was one reported complication and no reported mortality [20].

Gory et al. conducted a multicenter retrospective cohort study across multiple countries, including the United States, France, Italy, Germany, and Austria. Participants had a mean age of 62.89 years, and comorbidities such as diabetes mellitus, hypertension, cerebrovascular accident, and chronic kidney disease were included for the involvement of endovascular treatment with the PRANRD. Results showed 11 successful cases, which increased to 12 successful cases at the six-month follow-up. There were three reported complications, but no cases of mortality were observed in the study [19]. Detailed characteristics and outcomes of the included studies are summarized in Table 2.

**Table 2:** General outcomes The table summarizes key information from the studies included in the systematic review. Each study is identified by the author, title, country where it was conducted, and study design. The characteristics of the population, the intervention performed using PRANRD, the comparison intervention, and the characteristics of the evaluated aneurysms are described. A six-month follow-up was conducted and subsequently assessed with the RR scale and mRS. The number of cases with complications in each study is also shown. N/A: not applicable, PRANRD: PulseRider Aneurysm Neck Reconstructive Device, RR: Raymond-Roy, mRS: modified Rankin scale

Author, year	Limbucci et al., 2020 [18]	Mukherjee et al., 2017 [20]	Gory et al., 2017 [19]
Title	Y-stenting versus PulseRider-assisted coiling in the treatment of wide-neck bifurcation aneurysms: role of anatomical features on midterm results	PulseRider-assisted treatment of wide-necked intracranial bifurcation aneurysms: safety and feasibility study bifurcation aneurysms: safety and feasibility study	The PulseRider for the treatment of wide-neck bifurcation intracranial aneurysms: 6 months results
Country	Italy	United Kingdom	US center (Charleston, South Carolina, USA) European (Lyon and Besançon, France; Firenze and Treviso, Italy; Recklinghausen, Germany; Salzburg, Austria)
Study design	Cohort	Case series	Cohort
Age mean	56.47 ± 11.54	55	62.89
Cases age mean	58.88 ± 9.84	55	62.89
Control age mean	55.41 ± 12.12	55	62.89
Sex	Both	Both	Both
Intervention	PRANRD	PRANRD	PRANRD
Comparator	Y-stenting	N/A	N/A
Comorbidities	Hypertension	N/A	N/A
Follow-up time (months)	6	6	6
Mean dome size (mm)	N/A	N/A	8.83
Mean neck size (mm)	4.69	5.37	5.86
Dome-to-neck ratio (mm)	1.3	N/A	N/A
Total sample size	105	10	19
No. of cases	32	10	19
No. of controls	73	10	19
Cases RR1 (immediate)	22	10	11
Controls RR1 (immediate)	61	10	11
Cases RR1 (6 months)	20	10	12
Controls RR1 (6 months)	65	10	12
Cases RR2 (immediate)	9	N/A	6
Controls RR2 (immediate)	8	N/A	6
Cases RR2 (6 months)	7	N/A	6
Controls RR2 (6 months)	2	N/A	6
Cases RR3 (immediate)	1	N/A	2
Controls RR3 (immediate)	4	N/A	2
Cases RR3 (6 months)	5	N/A	1
Controls RR3 (6 months)	5	N/A	1
Cases mRS 0-2 at six months	N/A	3	N/A
Controls mRS 0-two at six months	N/A	3	N/A
Complications cases	2	1	N/A
Complication controls	6	1	N/A
Mortality cases	0	N/A	N/A
Mortality controls	1	N/A	N/A
Key points	The study aimed to compare the efficacy and safety of Y-stenting with PRANRD. Immediate adequate occlusion rates and complication rates were comparable between the Y-stenting and PRANRD groups, with figures of 94.5% and 96.9%, respectively, and 8.2% and 6.2%, respectively. At the six-month follow-up, adequate occlusion rates were 93.1% for Y-stenting and 84.3% for PRANRD. Complete occlusion rates were significantly higher with Y-stenting, showing 90.3% compared to 62.5% with PRANRD. Additionally, deterioration in occlusion grade was observed in 6.9% of Y-stenting patients versus 18.7% of PRANRD patients.	The study aimed to assess the safety and feasibility of PRANRD for intracranial WNBA. The study involved 10 patients who underwent PRANRD. Complete angiographic occlusion was achieved in all cases both immediately after the procedure and at the 6-month follow-up. There were no instances of procedural rupture or vessel dissection. Only one patient experienced nonocclusive thrombus formation, which did not lead to any clinical complications. At the 6-month follow-up, seven patients remained asymptomatic, while three patients had mild symptoms but were still able to perform their usual activities.	The study aimed to evaluate the outcomes of endovascular treatment for aneurysms, focusing on occlusion rates, morbidity, mortality, and recanalization rates at the six-month mark. The study demonstrated a high rate of adequate occlusion, accompanied by low morbidity and no mortality. Furthermore, the incidence of aneurysm recanalization at six months was found to be minimal.

Discussion

The WNBA is among the most challenging sacral aneurysms to treat [6,7]. Their particular anatomy makes surgical treatment challenging [8]. Nowadays, such challenging aneurysms can be treated safely with multiple endovascular techniques. However, comparing anatomical results remains difficult due to the varying characteristics of aneurysms. Despite the availability and efficacy of numerous treatments, new advances such as the PRANRD have emerged. This device is deployed at the parent vessel bifurcation across the aneurysm's neck, providing support within the framework of the aneurysm and creating a bridge to retain coils within the bulging artery. The device promotes early endothelialization while maintaining the coils in place with a minimal metal-to-artery ratio. Regardless of the anticipated efficacy of the PRANRD, our primary goal is to analyze the neurological complications that may arise with its use, as well as the stability and effectiveness of the device compared to the mortality rate.

The analysis of data from 134 patients across various geographic regions offers a comprehensive view of global practices and outcomes related to the PRANRD. Limbucci et al. compared the results regarding the efficacy and safety of Y-stenting versus PRANRD. The results showed immediate adequate occlusions, with complication rates being similar between the Y-stenting and PRANRD groups (94.5% vs. 96.9% and 8.2% vs. 6.2%, respectively). At a six-month follow-up, adequate occlusion rates were 93.1% after Y-stenting and 84.3% after PRANRD. Complete occlusion was significantly higher after Y-stenting (90.3% vs. 62.5% for PRANRD). Occlusion grade worsening occurred in 6.9% of Y-stenting patients and 18.7% of PRANRD patients, with only two complications reported for PRANRD and no mortality cases related to the device. Limbucci et al. concluded that Y-stenting and PRANRD are both effective and safe for treatment, with high immediate and mid-term occlusion rates [18].

Mukherjee et al. evaluated the safety and feasibility of PRANRD for intracranial wide-necked bifurcation aneurysms. The study included 10 patients who underwent PRANRD, achieving complete angiographic occlusion in all cases immediately post-procedure and at the six-month follow-up. No procedural ruptures or vessel dissections occurred, and only one case involved nonocclusive thrombus formation without clinical sequelae. At the six-month follow-up, seven patients were asymptomatic, while three had mild symptoms but were able to carry out their usual activities. With only one complication during the procedure and no reports of mortality, Mukherjee et al. stated that the use of PRANRD is safe and straightforward, as it is readily delivered in a standard method very similar to other available stents, making the procedure more familiar to operators new to the device [20].

Gory et al. studied the outcomes of endovascular treatment of aneurysms, focusing on occlusion rates, morbidity, mortality, and recanalization at six months. The study reported 11 successful cases with a high rate of adequate occlusion, three complications, low morbidity, and no mortality cases. Additionally, the risk of aneurysm recanalization at six months was low, indicating that PRANRD allows for effective aneurysm treatment with a high rate of adequate occlusion at six-month follow-ups [19].

The studies included in this systematic review indicate that PRANRD is effective and safe for WNBA. Other treatments mentioned in the studies, such as Y-stenting, have been accepted as a safe and reasonable alternative to clip reconstructions; however, its dense intravascular metal structure raises concerns about potential ischemia. Similarly, flow diversion stenting, which was recently introduced, is not ideal for treating bifurcations due to the potential for jailing the branching daughters and occluding the perforators. All these treatments require multiple steps to be performed successfully, and they are associated with higher morbidity and mortality rates. Given this information, PRANRD is technically easier to position and has been successful in reconstructing bifurcations with a low morbidity and mortality rate.

Despite the clear benefits that PRANRD provides in the short term and mid-term (six months), the long-term outcomes of this new device remain unknown. Complications such as permanent neurological deficits or total aneurysm occlusion have yet to be fully assessed over longer periods and in larger patient series. However, the low amount of metal, the device's positioning during the procedure, protection of the branching daughter vessels, and prevention of coil prolapse in the arterial occlusion suggest that PRANRD is a safe procedure with no procedure-related mortality.

The study faced several limitations, such as the lack of randomized controlled trials comparing PRANRD with other surgical procedures in larger patient series. Randomized controlled trials would help establish the differences between the PRANRD and other endovascular treatments used for aneurysms, facilitating the assessment of treatment outcomes and effectiveness. Moreover, randomized controlled trials are essential; their absence prevents us from evaluating the complications that patients might present beyond six months post-operation. A longer follow-up would provide a better understanding of the stability and safety of the new device. In addition, some of the included studies lacked complete demographic data, which limits the ability to fully assess patient characteristics and generalize the findings.

The short follow-up period was a significant limitation; a longer follow-up would have provided a better understanding of potential complications. Additionally, a notable limitation was the lack of comparative information on other surgical procedures using PRANRD.

## Conclusions

Our comprehensive study evaluated the efficacy, safety, and feasibility of employing the PRANRD compared to other interventions. The majority of the analyzed studies revealed favorable outcomes, significant long-term improvements, and minimal morbidity and mortality. The reviewed studies suggest PRANRD is a viable and safe option for treating WNBA. However, further research is essential to evaluate neurological complications and long-term outcomes.

Our findings support the safety and efficacy of the PRANRD as an endovascular treatment for WNBA. Given the device's safety and effectiveness, as demonstrated by sufficient aneurysm occlusion at a six-month follow-up and a low morbidity and mortality rate, additional research with a larger patient sample and extended follow-up is necessary. More data are required to substantiate its role as a viable endovascular treatment and to determine its long-term effectiveness beyond six months.
